# The role of mycobacteremia screening in enhancing non-tuberculous mycobacteria detection in hospitalized persons with HIV

**DOI:** 10.3389/fmicb.2025.1517418

**Published:** 2025-01-31

**Authors:** Mengjiao Miao, Hongyan Zhu, Ziyao Liu, Jinjin Yang, Yiting Zhang, Chenyu Ma, Jiamin Qin, Yaling Chen, Hongxia Wei, Wei Chen, Yongfeng Yang, Zhiliang Hu

**Affiliations:** ^1^Department of Infectious Disease, School of Public Health of Nanjing Medical University, The Second Hospital of Nanjing, Nanjing, China; ^2^Department of Infectious Disease, The Second Hospital of Nanjing, Nanjing University of Chinese Medicine, Nanjing, China; ^3^Clinical Research Center, The Second Hospital of Nanjing, Affiliated to Nanjing University of Chinese Medicine, Nanjing, China; ^4^Center for Global Health, School of Public Health, Nanjing Medical University, Nanjing, China

**Keywords:** non-tuberculous mycobacteria, mycobacteremia, HIV, mycobacterial avium complex, screening

## Abstract

Among hospitalized severely immunocompromised persons with HIV (PWH), non-tuberculous mycobacteria (NTM) may be under-diagnosed due to non-specific symptoms undifferentiable with other opportunistic infections. To evaluate the prevalence of NTM mycobacteremia and how screening for mycobacteremia assistant with identification of NTM infections, this study retrospectively analyzed 1,136 hospitalized PWH with CD4 counts <200 cells/μL, who underwent mycobacteremia screening at a tertiary hospital in Nanjing, China, between July 2018 and December 2023. The prevalence of non-tuberculous mycobacteremia was 5.8% (95% CI, 4.6–7.3%), with a higher prevalence of 8.6% (95% CI, 6.7–10.9%) in patients with CD4 counts <50 cells/μL, compared to 1.4% (95% CI, 0.6–3.0%) in those with CD4 counts ≥50 cells/μL. Mycobacterium avium complex (MAC) was the predominant pathogen, representing 95% (95% CI, 86.7–98.3%) of positive blood cultures, though it accounted for only 66.2% (95% CI, 54.3–76.3%) of NTM species isolated from respiratory samples. The sensitivity of mycobacterial blood cultures (MBC) in diagnosing all culture-proven NTM infections was 63.9% (95% CI, 54.0–72.8%), increasing to 75.7% (95% CI, 64.8–84.0%) in patients with CD4 counts <50 cells/μL. Notably, mycobacteremia served as the sole microbiological evidence in approximately 25% of all culture-proven NTM infections during initial hospitalization, where other specimen sources failed to yield conclusive evidence. These findings underscore the importance of mycobacteremia screening in improving the detection of NTM infections among severely immunocompromised hospitalized patients, especially those with CD4 counts <50 cells/μL, and highlight the value of incorporating MBC into diagnostic protocols to enhance clinical management of these high-risk individuals.

## 1 Introduction

Non-tuberculous mycobacteria (NTM) are ubiquitous environmental organisms found in soil and water, capable of causing a spectrum of diseases in humans. While NTM primarily results in pulmonary infections among the immunocompetent, such as those with underlying lung conditions like chronic obstructive pulmonary disease or bronchiectasis (Johansen et al., 2020; Gopalaswamy et al., 2020), the scenario is markedly different in the context of HIV. In severely immunocompromised persons with HIV (PWH), NTM infections frequently present as disseminated diseases, complicating the clinical landscape by blurring the lines with other opportunistic infections prevalent in this population (Yuan et al., 2023; Mickymaray et al., 2020; Nelson et al., 2023). The atypical manifestations and the often-disseminated nature of NTM in advanced HIV pose significant diagnostic challenges, leading to potential misdiagnoses and delayed interventions which could adversely affect patient prognosis. Studies suggest that the prognosis for PWH with disseminated NTM infections remains poor, with a substantial mortality rate even in regions with relatively robust healthcare systems (Hu et al., 2022; Kobayashi et al., 2016).

The inherent difficulties in diagnosing disseminated NTM infections underscore the need for effective screening strategies that can facilitate early detection. Blood-based mycobacterial detection offers a promising diagnostic approach due to its practicality and relative non-invasiveness. Screening for mycobacteremia at the point of hospital admission could potentially enable earlier diagnosis of disseminated NTM infections, thereby improving clinical outcomes. This study aims to retrospectively analyze the prevalence of mycobacteremia among hospitalized PWH at a tertiary specialty hospital in Nanjing, China, to assess how effectively mycobacteremia screening can aid in diagnosing NTM infections, and to identify those at the highest risk for disseminated NTM infections. By determining the prevalence and examining the diagnostic utility of mycobacterial blood screening, this research seeks to refine current screening protocols and enhance the management of NTM diseases in this high-risk group.

## 2 Materials and methods

### 2.1 Study design and participants

This retrospective observational study was carried out in the Department of Infectious Diseases at the Second Hospital of Nanjing, China. The setting is a tertiary referral hospital designated for providing comprehensive HIV care and is designated as a regional medical center for infectious diseases in Jiangsu Province, China. The facility is an integral part of the healthcare system in Nanjing, an area with relatively abundant medical resources.

As part of the standard protocol established by an expert panel in the department, all hospitalized patients with HIV (PWH) who had CD4 counts less than 200 cells/μL underwent routine diagnostic measures tailored to their specific clinical conditions. Independent of the presence of clinical symptoms, it was recommended to screen for tuberculosis via an interferon-gamma release assay (IGRA), for cryptococcal disease via serum cryptococcal antigen testing, and for cytomegalovirus (CMV) reactivation through blood CMV DNA load assessments. Following a departmental guideline update in 2018, screening for mycobacterial bacteremia in this population was advised though not mandated, leaving the final decision to perform mycobacterial blood cultures to the discretion of the attending clinicians.

Our previous research has highlighted the diagnostic and prognostic value of screening for cryptococcal antigenemia and blood CMV load in hospitalized PWH with severe immunodeficiency (Fang et al., 2023; Xu et al., 2020). The present study extended these investigations by retrospectively analyzing clinical data from PWH aged at least 18 years admitted to the infectious diseases unit from July 2018 to December 2023, with CD4 counts below 200 cells/μL and who were screened for mycobacterial bacteremia. We collected data on demographics, underlying diseases, clinical manifestations, laboratory tests, and diagnosis and treatment details from an electronic health record system. The study was approved by the Ethics Committee of the Second Hospital of Nanjing, and the requirement for informed consent was waived by the Ethics Committee.

### 2.2 Mycobacterial blood culture

The mycobacterial blood culture process encompassed several critical steps using the BD BACTEC^™^ MGIT^™^ 960 system (Becton, Dickinson, and Company, NJ, United States). Initially, 3–5 mL of blood was collected in EDTA anticoagulant tubes. The blood was then mixed with an equal volume of 0.5% N-acetylcysteine, 3.0% NaOH, and 1.45% sodium citrate solution. This mixture was vortexed and incubated at room temperature for 15 min. Subsequently, 40 mL of sterile phosphate-buffered saline was added, and the solution was thoroughly mixed and centrifuged at 3,000 g for 15 min, after which the supernatant was discarded. The pellet was inoculated into a liquid mycobacterial culture bottle and incubated using the BD BACTEC^™^ MGIT^™^ 960 system. If no growth of mycobacteria was detected after 42 days, the result was reported as negative. If the culture tests positive, a rapid colloidal gold-based diagnostic test (Hangzhou Creative Bio-Control Technology Co., Ltd., Hangzhou, China) was used to preliminary differentiate between *Mycobacterium tuberculosis* (MTB) and non-tuberculous mycobacteria (NTM) using the MPB64, which assists in guiding initial clinical treatment strategies. Further identification of the mycobacterial species was performed using a PCR-reverse dot blot method with a mycobacterial identification gene detection kit (Yaneng Bio, Shenzhen, China), which could distinguish MTB and 21 common pathogenic NTM species (Zhang et al., 2021; Abe et al., 1999).

### 2.3 Definitions of NTM diseases

Our diagnostic framework for NTM diseases in PWH incorporated established principles from guidelines by The American Thoracic Society (ATS), European Respiratory Society (ERS), European Society of Clinical Microbiology and Infectious Diseases (ESCMID), and Infectious Diseases Society of America (IDSA) (Daley et al., 2020). The guidelines emphasized a comprehensive evaluation encompassing clinical, radiographic, and microbiological criteria. Given the often disseminated and atypical manifestations of NTM infections in HIV patients (Nelson et al., 2023), we have adapted these guidelines to include advanced molecular diagnostic technologies such as next-generation sequencing (NGS)-based tests. This integration significantly enhanced our capability to identify NTM across various clinical contexts, aligning with the increasing use of NGS methods for the etiological diagnosis of infectious diseases, including mycobacterial infections (Wang and Xing, 2023; Zhu et al., 2022; Xiao et al., 2023; Zhang et al., 2023).

The diagnostic criteria for NTM diseases were tailored to address the unique challenges presented by HIV-associated NTM infections (Table 1). Criteria 1: Culture-proven NTM diseases, encompassed any culture-positive result for mycobacteria from clinical samples, such as bronchoalveolar lavage fluid, sputum, or biopsies, that are further identified as NTM. Criteria 2: Molecularly proven NTM diseases, applied when molecular diagnostics detected NTM-specific nucleic acids in clinical samples where a culture-proven diagnosis was not feasible. Criteria 3: Probable NTM diseases, applies when there was mycobacterial evidence but the specific NTM species cannot be definitively identified. Lastly, Criteria 4: Possible NTM diseases, was considered when the diagnosis was based solely on expert panel consultation without direct microbiological evidence.

**Table 1:** Definitions of NTM diseases.

Definitions	
Criteria 1: Culture-proven NTM diseases	
1A	Any sterile sample, BALF, or stool culture positive for mycobacteria and further identified as NTM
1B	Failed to satisfy 1A, at least two separate sputum samples culture positive for mycobacteria and further identified as NTM; or one sputum sample culture positive for mycobacteria and further identified as NTM combined with one positive sputum NTM molecular test
1C	Failed to satisfy 1A or 1B, one sputum sample culture positive for mycobacteria and further identified as NTM, and the expert panel concurred with the NTM diagnosis^a^
Criteria 2: Molecularly proven NTM diseases	
In the absence of mycobacterial culture results, if any clinical sample tests positive for NTM via molecular methods, an expert panel concurred with the NTM diagnosis	
Criteria 3: Probable NTM diseases	
3A	Failed to satisfy criteria 1 or criteria 2, mycobacterial blood culture positive without specific NTM species identification, and the expert panel concurred with the NTM diagnosis
3B	Failed to satisfy criteria 1, criteria 2 or 3A, AFB smears were positive, and the expert panel concurred with the NTM diagnosis
3C	Failed to satisfy criteria 1, criteria 2, 3A or 3B, the mycobacterial culture of a non-blood sample was positive without specific NTM species identification, and the expert panel concurred with the NTM diagnosis
Criteria 4: Possible NTM diseases	
The NTM diagnosis was based on the expert panel consultation without any microbiological evidence	

NTM, non-tuberculous mycobacteria; BALF, bronchoalveolar lavage fluid.

a The expert panel’s diagnosis of NTM was based on a comprehensive discussion of clinical and imaging findings.

### 2.4 Statistical analysis

Categorical variables were described as frequencies and proportions, while continuous variables were expressed by medians with interquartile ranges (IQR). Comparisons among different groups were done using the Mann–Whitney *U* test, chi-square test, or Fisher’s exact test, when appropriate. We calculated sensitivity, specificity, and positive and negative predictive values for each test. 95% CIs were calculated with the Wilson method for proportions. All reported *p*-values were two-sided, and the significance level was set at 0.05. All data were analyzed in R version 4.3.3.^1^

## 3 Results

### 3.1 Characteristics of the patients

**Figure 1:**
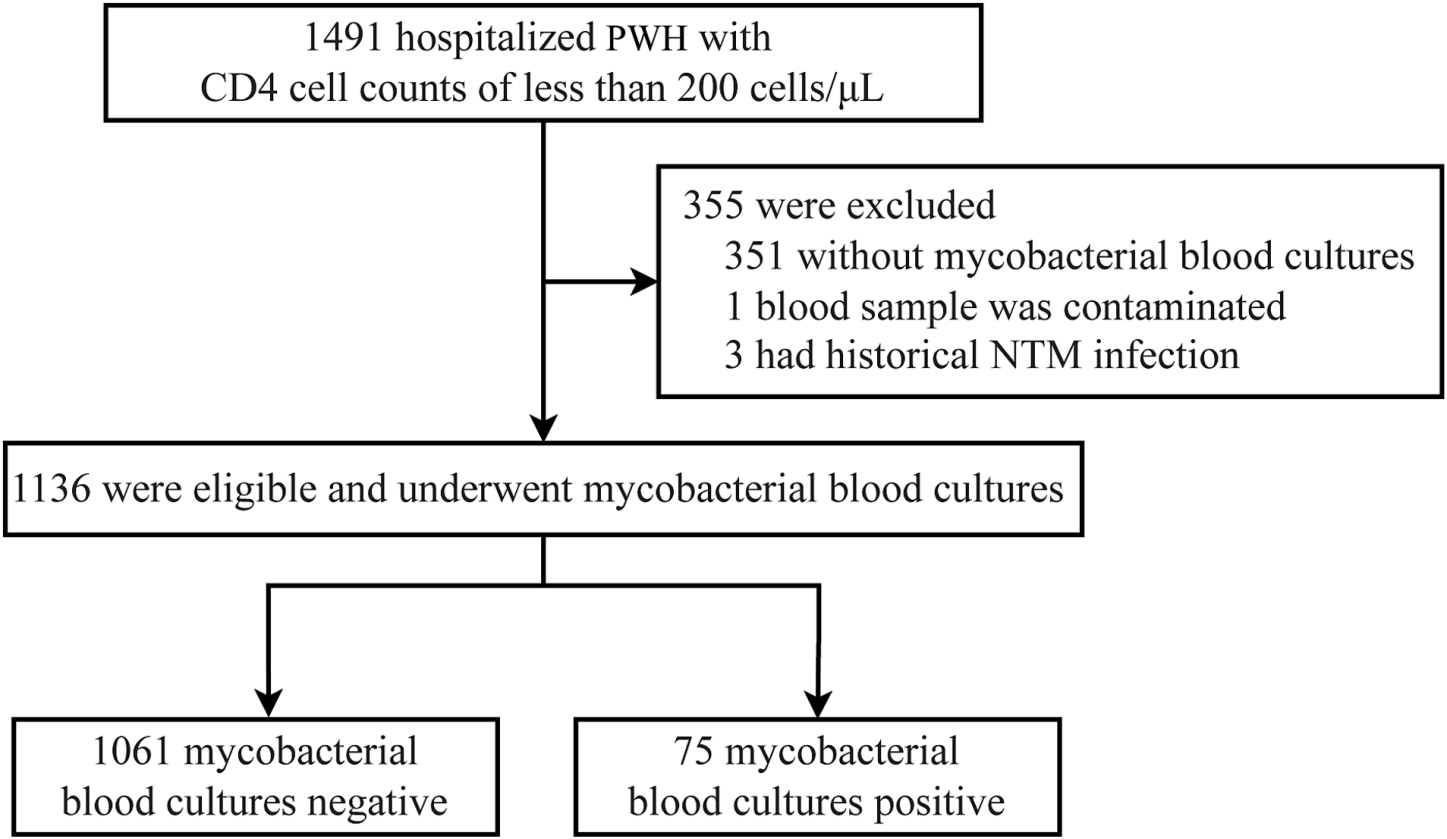
Flowchart of case selection process. PWH, persons with HIV; NTM, non-tuberculous mycobacteria.

Initially, the study considered 1,491 hospitalized persons with HIV (PWH) who had CD4 counts less than 200 cells/μL. Among these, 665 patients had CD4 counts ≥50 cells/μL, of whom 439 (66%) underwent screening for non-tuberculous mycobacteria (NTM). In the subset of patients with CD4 counts <50 cells/μL, a higher screening rate was observed, with 701 out of 826 patients (84.9%) being screened. Clinicians seemed to be more inclined to conduct this screening in patients with CD4 counts below 50 cells/μL (*p* < 0.001). After excluding 355 patients who did not undergo NTM screening, one patient due to suspected contamination of the blood sample, and three patients with a known history of NTM infection, a total of 1,136 PWH were included in the final analysis as depicted in Figure 1.

The characteristics of the patients were summarized in Table 2. The median age was 41 (IQR, 32–54) years, and 90.7% of the patients were male. About one-third of the patients had underlying diseases. In patients with other AIDS-defining illnesses (ADIs), pneumocystis pneumonia was the most common ADI. More than half of the patients experienced respiratory symptoms. Gastrointestinal symptoms occurred in 266 (23.4%) patients. At the time of admission, 977 (86.0%) patients were not on ART or only recently started ART. The median CD4 counts were 32 cells/μL (IQR, 11–74), and 699 (61.5%) patients had CD4 counts below 50 cells/μL. Of the 1,090 patients with available plasma HIV viral load results, the median HIV viral load was 5.12 log10 copies/mL (IQR, 4.33–5.62).

**Table 2:** Characteristics of 1,136 hospitalized PWH screened for mycobacteria.

Characteristics	Total (*n* = 1,136)	Mycobacterial blood culture		*p*-value
		Negative (*n* = 1,061)	Positive (*n* = 75)	
Age, years	41.0 (32.0, 54.0)	42.0 (32.0, 54.0)	33.0 (29.0, 44.0)	<0.001
18–59	990 (87.1)	918 (86.5)	72 (96.0)	0.028
≥60	146 (12.9)	143 (13.5)	3 (4.0)	
Sex				
Female	106 (9.3)	103 (9.7)	3 (4.0)	0.151
Male	1,030 (90.7)	958 (90.3)	72 (96.0)	
Underlying diseases	353 (31.1)	335 (31.6)	18 (24.0)	0.215
Hypertension	108 (9.5)	106 (10.0)	2 (2.7)	0.059
Coronary heart disease	26 (2.3)	26 (2.5)	0 (0.0)	0.331
Diabetes	86 (7.6)	81 (7.6)	5 (6.7)	0.936
Cerebrovascular disease	78 (6.9)	75 (7.1)	3 (4.0)	0.436
Chronic kidney disease	49 (4.3)	45 (4.2)	4 (5.3)	0.876
HBV co-infection	102 (9.0)	98 (9.2)	4 (5.3)	0.35
HCV co-infection	26 (2.3)	26 (2.5)	0 (0.0)	0.331
COVID-19	73 (6.4)	63 (5.9)	10 (13.3)	0.023
Other ADIs				
MTB	169 (14.9)	159 (15.0)	10 (13.3)	0.825
PCP	357 (31.4)	343 (32.3)	14 (18.7)	0.02
CMV retinitis	65 (5.7)	50 (4.7)	15 (20.0)	<0.001
Cryptococcal meningitis	55 (4.8)	53 (5.0)	2 (2.7)	0.529
Kaposi’s sarcoma	14 (1.2)	12 (1.1)	2 (2.7)	0.235
Lymphoma	38 (3.3)	37 (3.5)	1 (1.3)	0.503
Toxoplasma encephalitis	3 (0.3)	3 (0.3)	0 (0.0)	1
Respiratory symptoms	715 (62.9)	665 (62.7)	50 (66.7)	0.57
Gastrointestinal symptoms	266 (23.4)	241 (22.7)	25 (33.3)	0.05
Nausea and vomiting	127 (11.2)	121 (11.4)	6 (8.0)	0.475
Abdominal pain	71 (6.2)	62 (5.8)	9 (12.0)	0.06
Diarrhea	125 (11.0)	108 (10.2)	17 (22.7)	0.002
Hematochezia	25 (2.2)	22 (2.1)	3 (4.0)	0.489
ART status				<0.001
Not on ART or ART <12 weeks	977 (86.0)	914 (86.1)	63 (84.0)	
Effective ART ≥12 weeks	95 (8.4)	95 (9.0)	0 (0.0)	
Ineffective ART ≥12 weeks^a^	60 (5.3)	48 (4.5)	12 (16.0)	
ART ≥12 weeks, effectiveness not determined	4 (0.4)	4 (0.4)	0 (0.0)	
CD4 count, cells/μL	32 (11, 74)	36 (13, 79)	9 (4, 17)	<0.001
<50	699 (61.5)	632 (59.6)	67 (89.3)	<0.001
50–100	235 (20.7)	230 (21.7)	5 (6.7)	
100–200	202 (17.8)	199 (18.8)	3 (4.0)	
CD8 cell count, cells/μL	431 (235, 688)	445 (250, 705)	240 (132, 454)	<0.001
CD4/CD8 ratio	0.07 (0.03, 0.15)	0.08 (0.04, 0.15)	0.03 (0.01, 0.07)	<0.001
PVL, log10 copies/mL	5.12 (4.33, 5.62)	5.12 (4.33, 5.62)	5.08 (4.46, 5.63)	0.749
Missing data	46 (4.0)	43 (4.1)	3 (4.0)	

Data were shown as median (interquartile range, IQR) or *n* (%). *p*-values were derived from the Mann–Whitney *U* test, chi-square test or Fisher’s exact test as appropriate. HBV, hepatitis B virus; HCV, hepatitis C virus; COVID-19, coronavirus disease 2019; ADI, AIDS-defining illness; MTB, *Mycobacterium tuberculosis*; PCP, pneumocystis pneumonia; CMV, cytomegalovirus; ART, antiretroviral therapy; PVL, plasma HIV viral load.

a “Ineffective ART ≥12 weeks” refers to cases where patients have been continuously receiving antiretroviral therapy for more than 12 weeks, yet exhibit uncontrolled viral loads, suggesting potential resistance to the treatment.

### 3.2 Prevalence of mycobacteremia in hospitalized PWH

**Figure 2:**
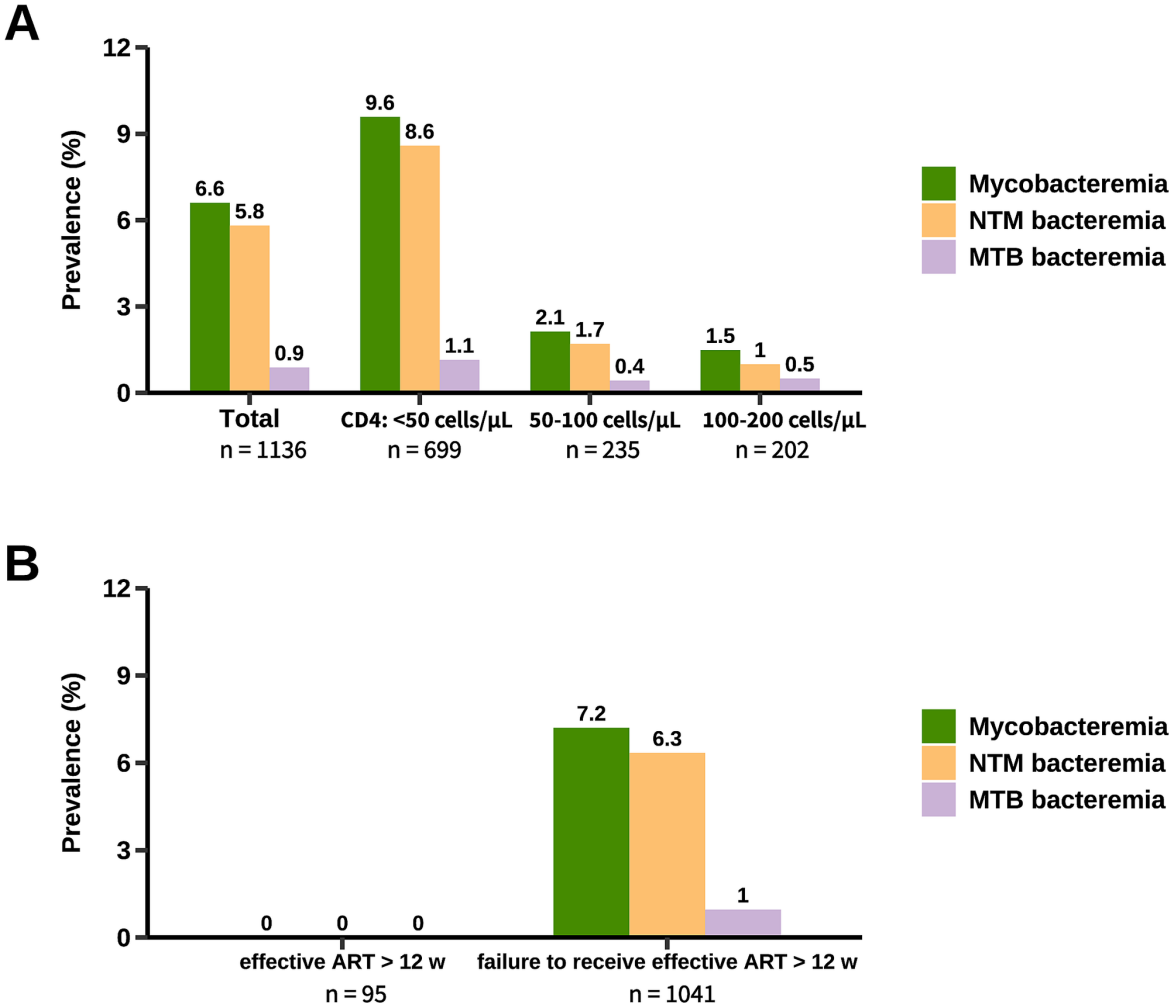
Prevalence of mycobacteremia in hospitalized PWH. The prevalence of mycobacteremia was stratified by CD4 count **(A)** and ART status **(B)**. Patients categorized under “Failure to receive effective ART for more than 12 weeks” included those not currently on ART, those who had been on ART for less than 12 weeks, and those on ART for more than 12 weeks but with treatment considered ineffective. NTM, non-tuberculous mycobacteria; MTB, *Mycobacterium tuberculosis*; ART, antiretroviral therapy.

The prevalence of mycobacteremia in the studied population was 6.6% (95% CI, 5.3–8.2%) (Figure 2A). Patients with mycobacteremia were younger, more likely to have CMV end-organ disease and gastrointestinal symptoms, and had lower CD4 counts (Table 2). When stratified by CD4 count, the prevalence of mycobacteremia was 9.6% (95% CI, 7.6–12.0%) in patients with CD4 counts below 50 cells/μL, 2.1% (95% CI, 0.9–4.9%) in those with CD4 counts between 50 to 100 cells/μL, and 1.5% (95% CI, 0.5–4.3%) in those with CD4 counts between 100 to 200 cells/μL. Of the 75 patients with mycobacteremia, mycobacterial species were successfully identified in 71 (94.7%) patients, with the vast majority being NTM species (87.3%; 62/71). The remaining 4 (5.3%; 4/75) patients in whom mycobacterial species could not be identified were classified as probable NTM diseases according to criteria 3 in the definitions of NTM diseases and were included in the analysis of the prevalence of NTM mycobacteremia (Table 1).

The prevalence of mycobacteremia caused by NTM and MTB was 5.8% (95% CI, 4.6–7.3%) and 0.9% (95% CI, 0.5–1.6%), respectively. For patients with CD4 counts below 50 cells/μL, the prevalence of NTM mycobacteremia and MTB mycobacteremia was 8.6% (95% CI, 6.7–10.9%) and 1.1% (95% CI, 0.6–2.2%), respectively (Figure 2A). Non-tuberculous mycobacteremia was only identified in 1.4% (95% CI, 0.6–3.0%) of the patients with CD4 count ≥50 cells/μL. Notably, among patients effectively treated with ART for more than 12 weeks, screening for mycobacteremia yielded no positive results (Figure 2B).

### 3.3 Distribution of NTM subspecies

**Figure 3:**
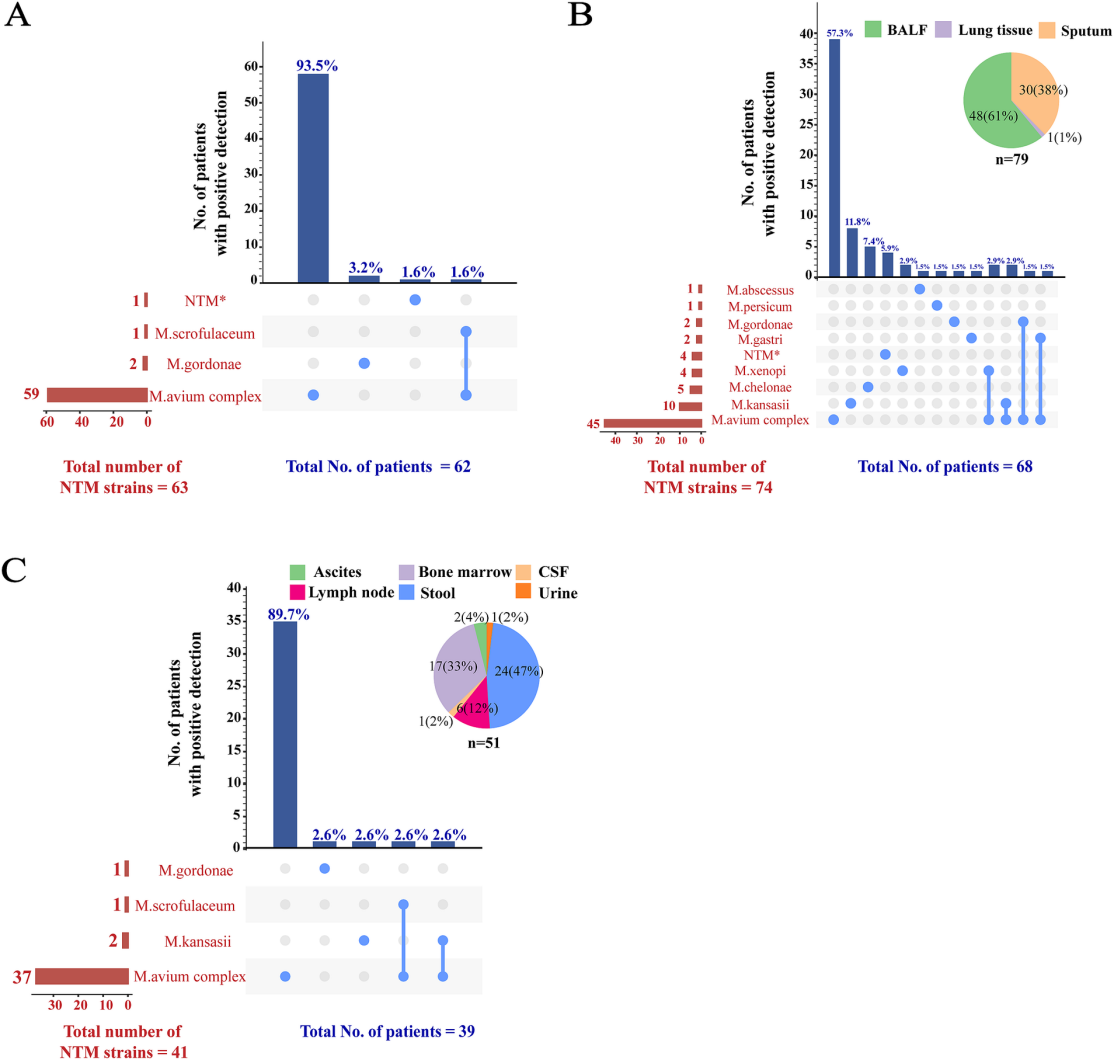
Distribution of NTM subspecies among different samples. The distribution of NTM subspecies among blood, respiratory, and other samples was illustrated in panels **(A–C)**, respectively. To demonstrate instances of mixed infections within the same patient and to concurrently display the detection summary for each strain, a matrix format was employed to present the data along the *x*-axis of the bar charts. In this matrix, rows represent NTM species, while columns correspond to the NTM detection patterns in individual patients. Columns containing a single NTM species indicated a solitary infection; columns with multiple NTM species suggested mixed infections. The sample types in panels **(B,C)** were represented using pie charts. ^*^MPB64 protein test was negative indicating an NTM strain, although subsequent genotyping result were unavailable. NTM, non-tuberculous mycobacteria; BALF, bronchoalveolar lavage fluid; CSF, cerebrospinal fluid.

For the 62 patients whose mycobacterial blood cultures grew NTM species, 59 (95.2%) had MAC mycobacteremia, *M. gordonae* and *M. scrofulaceum* occasionally caused blood dissemination. Isolation of multiple NTM species in blood samples was rare (Figure 3A). The distribution of NTM subspecies detected in other samples differed significantly from that in the bloodstream. Among the 68 patients with NTM identified from respiratory samples, the NTM subspecies were more diverse (Figure 3B). The four most frequently identified NTM subspecies were *M. avium* complex (66.2%; 45/68), *M. kansasii* (14.7%; 10/68), *M. chelonae* (7.4%; 5/68), and *M. xenopi* (5.9%; 4/68). Overall, these four NTM subspecies were detected in 88.2% of the 68 patients with NTM identified from respiratory samples. Simultaneous identification of two NTM subspecies was observed in 6 (8.8%; 6/68) patients. The distribution of NTM subspecies in non-bloodstream extrapulmonary infection resembled that in bloodstream infection, with MAC being the most commonly detected NTM subspecies (94.9%; 37/39) (Figure 3C).

### 3.4 Diagnostic performance of mycobacterial blood culture for NTM diseases

Gold standard cases could not be defined according to the case definitions (Table 1) in four patients (detailed information was summarized in Supplementary Table S1) who had a positive mycobacterial blood culture result returned after discharge and did not return for further mycobacterial subspecies classification. Therefore, these four patients were excluded from the diagnostic performance analysis. Overall, 194 (17.1%; 194/1,132) patients were diagnosed with NTM diseases, including 97 (8.6%; 97/1,132) cases of culture-proven NTM. The distribution of NTM diseases, as defined by the case definitions in Table 1, was summarized in Table 3. The diagnostic performance of mycobacterial blood culture for NTM diseases stratified by different case definitions was summarized in Table 4.

**Table 3:** Numbers of NTM diseases.

Criteria	Number of patients (*n*/*N*)
Criteria 1: Culture-proven NTM diseases	97/1,136 (8.5%)
1A	88/1,136 (7.7%)
1B	6/1,136 (0.5%)
1C	3/1,136 (0.3%)
Criteria 2: Molecularly proven NTM diseases	22/1,136 (1.9%)
Criteria 3: Probable NTM diseases	10/1,136 (0.7%)
3A	4/1,136 (0.4%)
3B	3/1,136 (0.3%)
3C	3/1,136 (0.3%)
Criteria 4: Possible NTM diseases	65/1,136 (5.7%)
Total	194/1,136 (17.1%)

NTM, non-tuberculous mycobacteria.

**Table 4:** Performance of mycobacterial blood culture for diagnosis NTM disease.

	Sensitivity (95% CI; *n*/*N*)	Specificity (95% CI; *n*/*N*)	PPV (95% CI; *n*/*N*)	NPV (95% CI; *n*/*N*)
Criteria 1	63.9 (54–72.8; 62/97)	100 (99.6–100; 1,035/1,035)	100 (94.2–100; 62/62)	96.7 (95.5–97.6; 1,035/1,070)
CD4 <50 cells/μL	75.7 (64.8–84; 56/74)	100 (99.4–100; 621/621)	100 (93.6–100; 56/56)	97.2 (95.6–98.2; 621/639)
CD4: 50–200 cells/μL	26.1 (12.5–46.5; 6/23)	100 (99.1–100; 414/414)	100 (61–100; 6/6)	96.1 (93.8–97.5; 414/431)
Criteria 1 + 2	52.1 (43.2–60.9; 62/119)	100 (99.6–100; 1,013/1,013)	100 (94.2–100; 62/62)	94.7 (93.2–95.9; 1,013/1,070)
CD4 <50 cells/μL	63.6 (53.2–72.9; 56/88)	100 (99.4–100; 607/607)	100 (93.6–100; 56/56)	95 (93–96.4; 607/639)
CD4: 50–200 cells/μL	19.4 (9.2–36.3; 6/31)	100 (99.1–100; 406/406)	100 (61–100; 6/6)	94.2 (91.6–96; 406/431)
Criteria 1 + 2 + 3	49.6 (41–58.2; 62/125)	100 (99.6–100; 1,007/1,007)	100 (94.2–100; 62/62)	94.1 (92.5–95.4; 1,007/1,070)
CD4 <50 cells/μL	60.9 (50.7–70.2; 56/92)	100 (99.4–100; 603/603)	100 (93.6–100; 56/56)	94.4 (92.3–95.9; 603/639)
CD4: 50–200 cells/μL	18.2 (8.6–34.4; 6/33)	100 (99.1–100; 404/404)	100 (61–100; 6/6)	93.7 (91–95.7; 404/431)
Criteria 1 + 2 + 3 + 4	32.6 (26.4–39.6; 62/190)	100 (99.6–100; 942/942)	100 (94.2–100; 62/62)	88 (86–89.8; 942/1,070)
CD4 <50 cells/μL	41.2 (33.3–49.6; 56/136)	100 (99.3–100; 559/559)	100 (93.6–100; 56/56)	87.5 (84.7–89.8; 559/639)
CD4: 50–200 cells/μL	11.1 (5.2–22.2; 6/54)	100 (99–100; 383/383)	100 (61–100; 6/6)	88.9 (85.5–91.5; 383/431)

Criteria 1: Culture-proven cases of NTM. Criteria 1 + 2: Culture and molecularly proven cases of NTM. Criteria 1 + 2 + 3: Culture and molecularly proven cases of NTM plus probable cases of NTM. Criteria 1 + 2 + 3 + 4: Culture and molecularly proven cases of NTM plus probable and possible cases of NTM. PPV, positive predictive value; NPV, negative predictive value; CI, confidence interval.

The sensitivity, specificity, positive predictive value (PPV), and negative predictive value (NPV) of mycobacterial blood culture for diagnosing culture-proven NTM cases were 63.9% (95% CI, 54–72.8%), 100% (95% CI, 99.6–100%), 100% (95% CI, 94.2–100%), and 96.7% (95% CI, 95.5–97.6%), respectively. In patients with CD4 counts below 50 cells/uL, the sensitivity and NPV increased to 75.7% (95% CI, 64.8–84.0%) and 97.2% (95% CI, 95.6–98.2%), respectively. For diagnosing all proven NTM cases, the sensitivity, specificity, PPV, and NPV of mycobacterial blood culture were 52.1% (95% CI, 43.2–60.9%), 100.0% (95% CI, 99.6–100%), 100.0% (95% CI, 94.2–100%), and 94.7% (95% CI, 93.2–95.9%), respectively. The sensitively and NPV increased to 63.6% (95% CI, 53.2–72.9%; 56/88) and 95% (95% CI, 93–96.4%; 607/639), respectively, in patients with CD4 counts below 50 cells/uL.

### 3.5 Impact of mycobacteremia screening on NTM case identification

**Figure 4:**
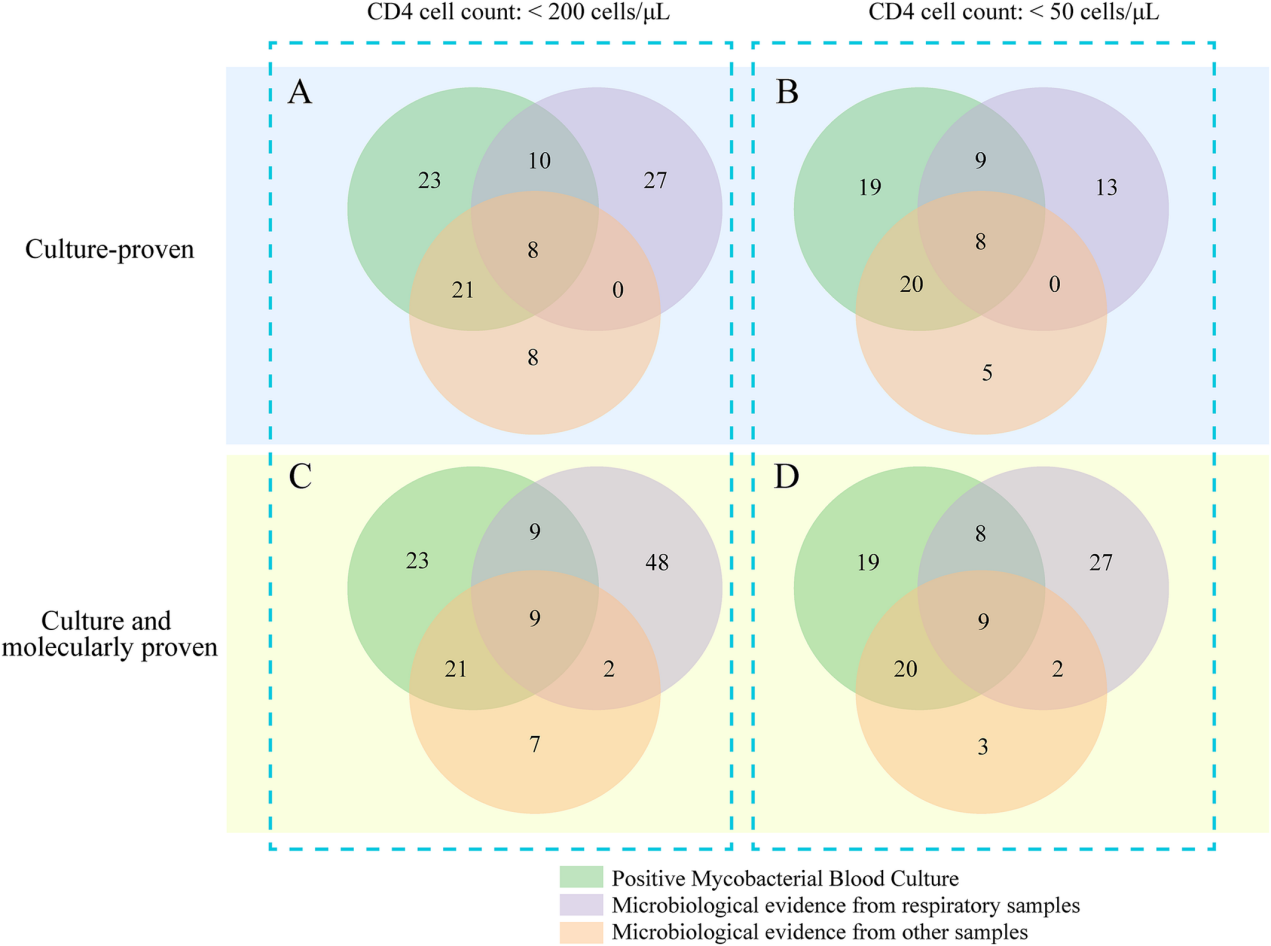
Venn diagram illustrating the distribution of positive test results across different sample types.

Among the 97 culture-proven NTM cases, mycobacterial blood cultures provided the sole microbiological evidence in 23 patients (23.7%; 95% CI: 16.4–33.1%) during their initial hospitalization, where other specimen sources failed to yield conclusive evidence of NTM infection (Figure 4). Overall, mycobacteremia screening contributed to two distinct findings: it served as the only microbiological clue for 12 patients (12.4%; 95% CI: 7.1–20.6%) in whom NTM infection was clinically suspected, and it unexpectedly identified 11 cases (11.3%; 95% CI: 6.2–19.2%) in which NTM infection was not initially considered by the clinical team.

In the subgroup of 74 patients with CD4 counts <50 cells/μL, blood mycobacterial cultures provided the sole diagnostic evidence for 19 patients (25.7%; 95% CI: 17.1–36.7%) whose other specimen sources were non-diagnostic. The use of advanced molecular diagnostics further identified 21 additional pulmonary NTM cases and one case of central nervous system NTM disease, underscoring the importance of complementary diagnostic strategies. Among the 119 total NTM cases identified by either culture or molecular methods, mycobacteremia was the only microbiological finding in 19.3% of cases (95% CI: 13.2–27.3%) during the initial hospital stay (Figure 4).

## 4 Discussion

NTM infections remain a significant opportunistic infection among late-stage PWH, yet the reported prevalence varies substantially across different countries and healthcare settings. In the United States, NTM diseases account for approximately 12% of the cases among PWH hospitalized due to opportunistic infections (Bielick et al., 2024). In contrast, while high rates of tuberculosis diagnosis are reported among PWH in West Africa, NTM diseases are rarely diagnosed (Lewden et al., 2014). In China, the reported prevalence of NTM diseases among hospitalized PWH fluctuates between 2.2 and 11.3% (Luo et al., 2016; Xiao et al., 2013; Pang et al., 2018). This variation in reported rates of hospital NTM prevalence may be attributed to differences in patient demographics at various medical facilities, selection biases inherent in hospital-based epidemiological studies, or disparities in the diagnostic capabilities for NTM pathogens across different settings. Another potential factor is the lack of characteristic manifestations in PWH co-infected with NTM (Nelson et al., 2023), which, if not routinely screened for, may lead to underdiagnosis of the condition. Despite the significance of NTM as a critical opportunistic infection in PWH, current guidelines do not provide recommendations for routine screening of MAC infections, nor specify which populations should be screened or how to screen for MAC infections, primarily due to the limited real-world data supporting such a screening strategy.

In this study, conducted in a higher-resource healthcare setting that manages PWH, it was found that systematic screening for key opportunistic infections, including NTM through blood culture, is vital for enhancing survival among hospitalized late-stage PWH. The data revealed that the prevalence of non-tuberculous mycobacteremia among hospitalized PWH with CD4 counts below 200 cells/μL stood at 5.8% (95% CI, 4.6–7.3%). More critically, the prevalence escalated to 8.6% (95% CI, 6.7–10.9%) among those with CD4 counts less than 50 cells/μL, in stark contrast to a mere 1.4% (95% CI, 0.6–3.0%) in patients with CD4 counts ≥50 cells/μL. This marked disparity underscores the necessity of NTM bacteremia screening within this vulnerable subgroup to potentially improve clinical outcomes.

Previous studies have established that the majority of NTM species isolated from the blood of PWH are predominantly from the MAC (Lopez-Luis et al., 2020; Varley et al., 2017). Echoing these observations, our current study identified MAC as the dominant NTM species, accounting for approximately 95% of isolates from both blood and extrapulmonary specimens. The notable prevalence of MAC in blood samples does not imply that late-stage HIV patients are exclusively susceptible to MAC infections, as in our study only about two-thirds of NTM isolated from respiratory specimens were MAC. This indicates that MAC may have enhanced capabilities for hematogenous invasion under conditions of compromised immunity. Future research should consider employing MAC-specific molecular diagnostic techniques to enhance the detection of NTM bacteremia. Furthermore, our findings reveal that in hospitalized PWH with CD4 counts below 50 cells/uL, reliance solely on cultures from respiratory specimens risks missing over half of the culture-proven NTM infections (Figure 4). Incorporating screening for bacteremia identified 75.7% (95% CI, 64.8–84.0%) of culture-proven NTM infections in this subgroup. Notably, in nearly a quarter of these cases, mycobacteremia was the sole clinical indicator of NTM infection. These results highlight that MBC not only demonstrates acceptable sensitivity for diagnosing NTM diseases in severely immunocompromised hospitalized PWH but also plays a crucial role in uncovering NTM infections that other specimen tests might overlook.

Primary prophylaxis for disseminated MAC is recommended for PWH with a CD4 count below 50 cells/μL who are either not on ART or are on ART but not achieving viral suppression. The relatively high prevalence of NTM bacteremia observed in our study within such groups substantiates the need for primary prophylaxis in individuals at high risk for disseminated NTM infections. Importantly, while prophylactic measures are essential, caution is warranted due to the potential for developing resistance if prophylaxis is administered to patients with existing NTM bacteremia. Therefore, confirming the absence of active disseminated MAC infection prior to starting prophylaxis is recommended. The guidelines further clarify that prophylaxis should be reserved for those individuals who are not effectively managed with ART. Notably, our findings reveal that patients who have been stably treated with ART for more than 12 weeks exhibited no NTM bacteremia upon hospital admission, highlighting the crucial role of timely and effective ART in preventing such late-stage opportunistic infections among persons with HIV.

Our study has several limitations. First, within the subgroup of hospitalized PWH with CD4 counts below 200 cells/μL, the expert panel in our medical setting only recommends, rather than mandates, screening for NTM bacteremia. Although the majority of patients with CD4 counts below 50 cells/μL completed the NTM bacteremia screening, only two-thirds of those with CD4 counts of 50 cells/μL or higher did so. Cases not screened were likely considered by clinicians to have a low probability of disseminated NTM bacteremia. Consequently, the prevalence of NTM bacteremia in patients with CD4 counts above 50 cells/μL might be overestimated. Secondly, our study focused solely on hospitalized patients. Those admitted are likely to have more severe or advanced disease compared to the outpatient population. Therefore, it is inappropriate to generalize the NTM bacteremia prevalence data to PWH in an outpatient setting. Lastly, since our study is a single-center investigation, the findings might be influenced by regional healthcare practices and other localized factors.

## 5 Conclusion

In conclusion, non-tuberculous mycobacteremia is moderately prevalent among hospitalized PWH with CD4 counts <50 cells/μL, with MAC being the most prevalent pathogen. Mycobacteremia screening plays a critical role in improving the detection of NTM infections, providing essential microbiological evidence when other sources fail. This study highlights the importance of including mycobacterial blood cultures in diagnostic protocols for severely immunocompromised patients to enhance early and accurate identification of NTM infections.

## Data Availability

The raw data supporting the conclusions of this article will be made available by the authors, without undue reservation.

## Footnotes

^1^ www.r-project.org

## Glossary

ADIs: AIDS-defining illnesses
ART: Antiretroviral therapy
ATS: American Thoracic Society
BALF: Bronchoalveolar lavage fluid
CI: Confidence interval
CMV: Cytomegalovirus
COVID-19: Coronavirus disease 2019
CSF: Cerebrospinal fluid
ERS: European Respiratory Society
ESCMID: European Society of Clinical Microbiology and Infectious Diseases
HBV: Hepatitis B virus
HCV: Hepatitis C virus
HIV: Human immunodeficiency virus
IDSA: Infectious Diseases Society of America
IGRA: Interferon-gamma release assay
IQR: Interquartile ranges
MAC: Mycobacterial avium complex
MBC: Mycobacterial blood culture
MTB: *Mycobacterium tuberculosis*
NGS: Next-generation sequencing
NPV: Negative predictive value
NTM: Non-tuberculous mycobacteria
PCP: Pneumocystis pneumonia
PPV: Positive predictive value
PVL: Plasma HIV viral load
PWH: Persons with HIV
TB: Tuberculosis
