# Supplementary material for: The role of mycobacteremia screening in enhancing non-tuberculous mycobacteria detection in hospitalized persons with HIV
Source: Front Microbiol. 2025 Jan 31;16:1517418. doi: 10.3389/fmicb.2025.1517418 (PMC11825463; doi:10.3389/fmicb.2025.1517418)
Supplement: Supplementary file 1 [file Table_1.DOCX]

| **Definitions** | |
| --- | --- |
| **Criteria 1: Culture-proven NTM diseases** | |
| 1A | Any sterile sample, BALF, or stool culture positive for mycobacteria and further identified as NTM. |
| 1B | Failed to satisfy 1A, at least two separate sputum samples culture positive for mycobacteria and further identified as NTM; or one sputum sample culture positive for mycobacteria and further identified as NTM combined with one positive sputum NTM molecular test. |
| 1C | Failed to satisfy 1A or 1B, one sputum sample culture positive for mycobacteria and further identified as NTM, and the expert panel concurred with the NTM diagnosis^1^. |
| **Criteria 2: Molecularly proven NTM diseases** | |
| In the absence of mycobacterial culture results, if any clinical sample tests positive for NTM via  molecular methods, an expert panel concurred with the NTM diagnosis. | |
| **Criteria 3: Probable NTM diseases** | |
| 3A | Failed to satisfy criteria 1 or criteria 2, mycobacterial blood culture positive without specific NTM species identification, and the expert panel concurred with the NTM diagnosis. |
| 3B | Failed to satisfy criteria 1, criteria 2 or 3A, AFB smears were positive, and the expert panel concurred with the NTM diagnosis. |
| 3C | Failed to satisfy criteria 1, criteria 2, 3A or 3B, the mycobacterial culture of a non-blood sample was positive without specific NTM species identification, and the expert panel concurred with the NTM diagnosis. |
| **Criteria 4: Possible NTM diseases** | |
| The NTM diagnosis was based on the expert panel consultation without any microbiological evidence. | |

**Table 1. Definitions of NTM Diseases**

NTM, nontuberculous mycobacteria; BALF, bronchoalveolar lavage fluid

^1^ The expert panel's diagnosis of NTM was based on a comprehensive discussion of clinical and imaging findings.
